# Supplementary material for: Resolving the full spectrum of human genome variation using Linked-Reads
Source: Genome Res. 2019 Apr;29(4):635–45. doi: 10.1101/gr.234443.118 (PMC6442396; doi:10.1101/gr.234443.118)
Supplement: Supplemental Material [file supp_gr.234443.118_Supplemental_longranger-2.2.zip › longranger-2.2.tar.bz2!/lr2.2-public/lib/stan/cmdstan/stan/doc/api/html/vvd__vari_8hpp_source.html]

Stan: src/stan/agrad/rev/internal/vvd\_vari.hpp Source File


|  |  |
| --- | --- |
|  | Stan  2.5.0  probability, sampling & optimization |


- Main Page
- Related Pages
- Namespaces
- Classes
- Files

- File List
- File Members

All Classes Namespaces Files Functions Variables Typedefs Enumerations Enumerator Friends Macros Pages

- src
- stan
- agrad
- rev
- internal

vvd\_vari.hpp

Go to the documentation of this file.

1 #ifndef STAN\_\_AGRAD\_\_REV\_\_INTERNAL\_\_VVD\_VARI\_HPP

2 #define STAN\_\_AGRAD\_\_REV\_\_INTERNAL\_\_VVD\_VARI\_HPP

3

4 #include <stan/agrad/rev/vari.hpp>

5

6 namespace stan {

7  namespace agrad {

8

9  class op\_vvd\_vari : public vari {

10  protected:

11  vari\* avi\_;

12  vari\* bvi\_;

13  double cd\_;

14  public:

15  op\_vvd\_vari(double f, vari\* avi, vari\* bvi, double c) :

16  vari(f),

17  avi\_(avi),

18  bvi\_(bvi),

19  cd\_(c) {

20  }

21  };

22

23  }

24 }

25 #endif

stan::agrad::op\_vvd\_vari

**Definition:** vvd\_vari.hpp:9

vari.hpp

stan::agrad::op\_vvd\_vari::op\_vvd\_vari

op\_vvd\_vari(double f, vari \*avi, vari \*bvi, double c)

**Definition:** vvd\_vari.hpp:15

stan::agrad::vari

The variable implementation base class.

**Definition:** vari.hpp:28

stan::agrad::op\_vvd\_vari::avi\_

vari \* avi\_

**Definition:** vvd\_vari.hpp:11

stan::agrad::op\_vvd\_vari::cd\_

double cd\_

**Definition:** vvd\_vari.hpp:13

stan::agrad::op\_vvd\_vari::bvi\_

vari \* bvi\_

**Definition:** vvd\_vari.hpp:12


---

[ Stan Home Page ]

*© 2011–2014,
Stan Development Team.*
